# Reankylosis of temporomandibular joint 5 years after interpositional arthroplasty using gold foil: management and follow up (a case report)

**DOI:** 10.1016/j.ijscr.2024.109311

**Published:** 2024-01-29

**Authors:** Poerwati Soetji Rahajoe, Panji Hendar Rismanto, M. Bakhrul Lutfianto

**Affiliations:** a Department of Oral and Maxillofacial Surgery, Faculty of Dentistry, Universitas Gadjah Mada, Yogyakarta, Indonesia; b Resident of Oral and Maxillofacial Surgery Study Program, Faculty of Dentistry, Universitas Gadjah Mada, Yogyakarta, Indonesia; c Oral and Maxillofacial Surgery Staff, Dr. Sardjito General Hospital, Yogyakarta, Indonesia

**Keywords:** Case report, Costochondral graft, Interpositional arthroplasty, Reankylosis, Temporalis muscle fascia

## Abstract

**Introduction:**

Temporomandibular Joint (TMJ) reankylosis is one of TMJ arthroplasty complications that can interfere masticatory function and aesthetics. This case report aimed to describe a TMJ reankylosis in growing age patient that occurred 5 years after interpositional arthroplasty using gold foil. Interpositional arthroplasty using temporalis fascia and costochondral graft followed by unilateral coronoidectomy could be a treatment option.

**Case presentation:**

A 17-year-old female came with inability to open her mouth 5 years after first interpositional arthroplasty using gold foil due to traumatic TMJ ankylosis. Patient was diagnosed type IV left TMJ reankylosis with left coronoid process hyperplasia. Patient was treated with interpositional arthroplasty using temporalis fascia as an interposition material for articular disc substitution, costochondral graft for ramus condyle unit (RCU) reconstruction and followed by unilateral coronoidectomy. Postoperative mouth opening was ±26 mm. One year evaluation showed stable mouth opening and no recurrency occured.

**Discussion:**

Age at growing period, insufficient gap width, surgical technique and the effects of previous surgery may generate TMJ reankylosis. Temporalis fascia widely used for interposision material and act as a lubricant that makes movement frictionless. Costochondral graft can be used for RCU reconstruction to prevent decreasing mandibular ramus height and openbite.

**Conclusion:**

Growing age increases the risk of TMJ reankylosis. Interpositional arthroplasty, which used temporalis fascia and a costochondral graft, has resulted in a sufficient mouth opening and an improvement in masticatory function. Recurrence was not found in the 1-year postoperative evaluation.

## Highlights

• Reankylosis of temporomandibular joint after interpositional arthroplasty using gold foil
• Growing age increases the risk of TMJ reankylosis
• Interpositional arthroplasty using temporalis muscle fascia and costochondral graft maybe a treatment option

## 1 Introduction

TMJ ankylosis is a condition of limited mouth opening because the mandibular condyle is fused with the glenoidal fossa by formation of fibrous, bone, or mixed tissue around the joint. It interferes with the process of mastication, speech, oral hygiene and facial symmetry [[1], [2], [3]].

Three most commonly used techniques for treating TMJ ankylosis include gap arthroplasty, interpositional arthroplasty, and total joint reconstruction [4]. TMJ arthroplasty may generate complication such as recurrence of ankylosis or reankylosis, which the incidency is 9–12 % in children and 2–7 % in adults [5].

TMJ reankylosis is triggered by inadequate gap, hematoma and bone dusts during cutting the bone to form a gap which has many osteoprogenitor cells which foster bone formation [6,7]. Age has become an influential factor in reankylosis because bone growth and remodeling are remarkably active during the age of growth [5,6] and in childhood age the patients cannot or will not cooperate towards postoperative instructions [6]. As a result, outcome is unsatisfactory. Treatment of TMJ reankylosis includes interpositional arthroplasty, total joint reconstruction and osteogenesis distraction [8,9]. Reankylosis has become a challenging treatment of TMJ ankylosis because it may end up in multiple surgeries.

This case report aimed to describe the management and follow up of a TMJ reankylosis in growing age patient that occurred 5 years after interpositional arthroplasty using gold foil which treated by second surgical procedure with interpositional arthroplasty using temporalis fascia and costochondral grafts followed by unilateral coronoidectomy.

## 2 Case presentation

### 2.1 Subjective and objective examinations

**Fig. 1:**
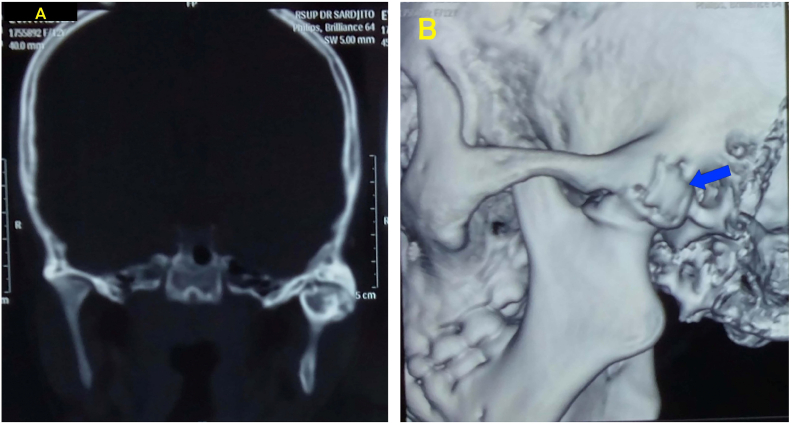
A and B Preoperative CT and 3D CT image before the first surgery, showed lateral and medial extensions of the ankylotic mass of left TMJ ankylosis (blue arrow). (For interpretation of the references to colour in this figure legend, the reader is referred to the web version of this article.)

**Fig. 2:**
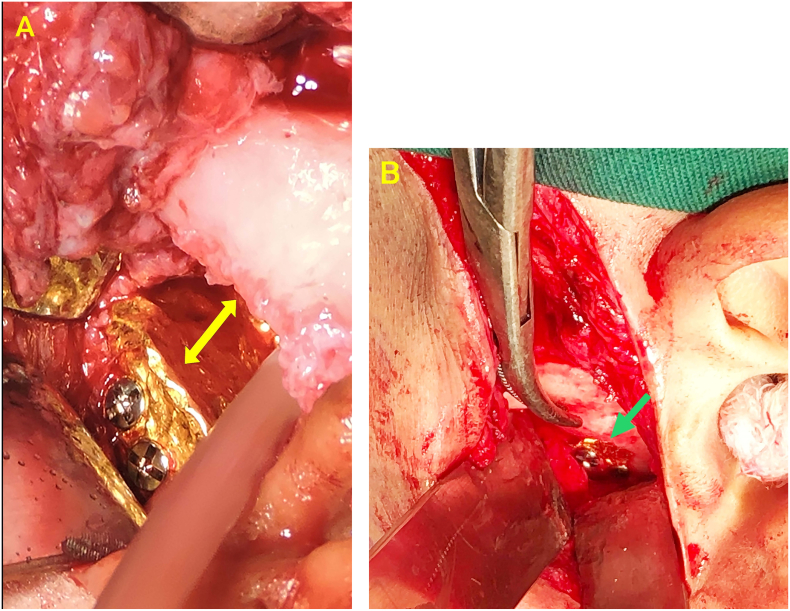
A The gold foil was used as an interposition material at the first surgery and the gap that has been created is visible (yellow arrow), B The second surgery showed that the gap has disappeared and was filled by bone (green arrow) and part of the gold foil was covered by bone. (For interpretation of the references to colour in this figure legend, the reader is referred to the web version of this article.)

A 17-year-old female patient came to the oral and maxillofacial surgery clinic of Sardjito Hospital with inability to open her mouth and difficulty to eat. The patient had a history of left TMJ ankylosis due to facial trauma (Fig. 1) which treated with interpositional arthroplasty using a gold foil 5 years ago (Fig. 2A). After surgery, the patient was able to open her mouth to 20 mm wide. Five years after surgery, the patient found symptoms of narrow mouth opening and inability to open her mouth.

The objective examination found the general condition within normal limits. Extraoral showed a light asymmetrical face. In a resting position left TMJ was palpable protrusion with a hard consistency and painless. Palpation of the TMJ in the mouth opening position found no movement nor pain. Mouth opening ability and mandibular movement towards the anterior and lateral were both 0 mm. Intraoral examination was not possible, leaving only buccal and labial side could be examined, and showed normal occlusion and good oral hygiene.

**Fig. 3:**
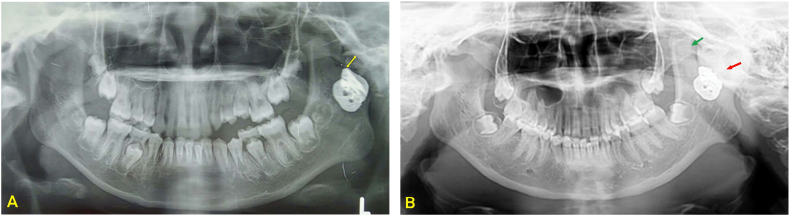
A Panoramic radiograph was taken immediately after the first arthroplasty. There was a radiopaque image of metal on the left mandibular ramus showing a gold foil as interposition material and there was a clear gap between the mandibular fossa and the ramus (yellow arrow), B Preoperative panoramic radiograph was taken 5 years after the first surgery. The gap between the mandibular fossa and ramus was not visible, filled by bone that extends over the mandibular incisura (red arrow). Hyperplasia or elongation of the left coronoid processus is visible (green arrow). (For interpretation of the references to colour in this figure legend, the reader is referred to the web version of this article.)

**Fig. 4:**
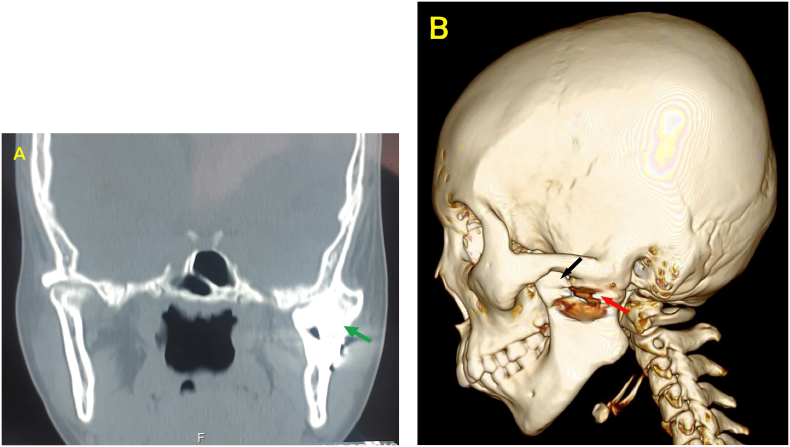
A CT image showed ankylotic bone mass, and there was no space between the glenoidal fossa and the left mandibular ramus (green arrow), B 3D CT image showed reankylosis which was characterized by bone growth between the glenoidal fossa and the left mandibular ramus. The gap has been closed by bone mass that extends covering the left mandibular incisura (red arrow). Hyperplasia left coronoid processus was visible (black arrow). (For interpretation of the references to colour in this figure legend, the reader is referred to the web version of this article.)

Panoramic radiograph exhibited internal fixation and metal sheet appearance on the left mandibular ramus (Fig. 3). Computed tomogram (CT) and 3-dimensional (3D) CT demonstrated narrowing of the joint space leading to a picture of left TMJ reankylosis (Fig. 4).

Based on the subjective, objective and supporting examinations, the patient was diagnosed with left TMJ reankylosis type IV (extensed ankylotic bone mass to the mandibular incisura) along with hyperplasia of the left coronoid process.

**Fig. 5:**
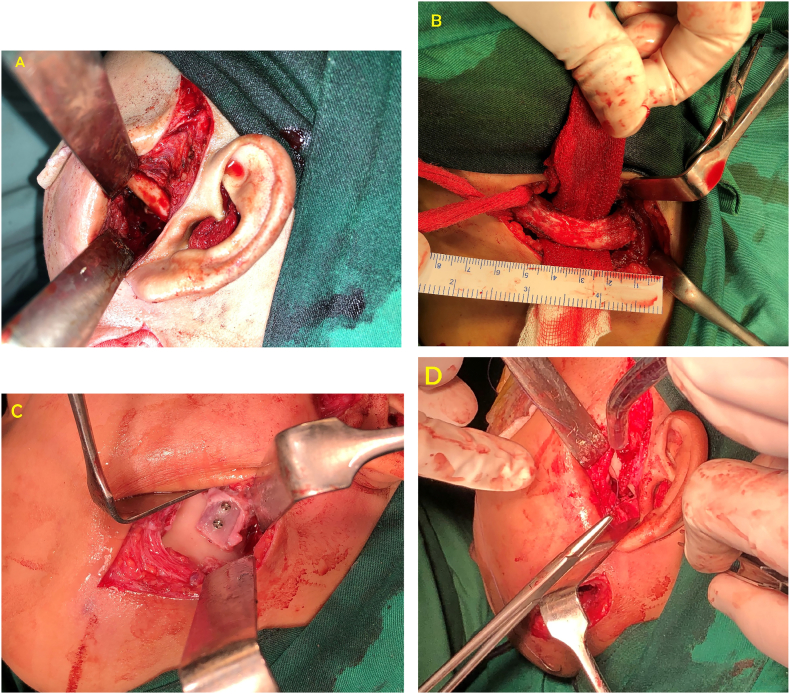
A 20 mm-wide gap is created, B Harvesting of costochondral graft by orthopedic surgeon, C Fixation of the costochondral graft using 2 screws, D Suture of the temporalis fascia flap on the tissue around the fixed costochondral graft area.

The second surgery (Fig. 5) was performed with interpositional arthroplasty using temporalis fascia and RCU reconstruction with a costochondral graft followed by unilateral coronoidectomy.

### 2.2 Surgical technique

The surgery was performed under general anesthesia involving an orthopedic surgeon to obtain costochondral graft. Intubation was performed consciously assisted with a fiberoptic endoscope as the patient failed to open her mouth.

Incision was done along the first surgical incision site and blunt dissection performed to avoid injury of facial nerve and temporalis artery. Upon reaching the bone, the ankylotic bone mass in the ramus region was seen extending to the mandibular incisura and glenoidal fossa. The gold foil was found fixed with two screws which is partially covered by the bone mass. No gap was found between the glenoid fossa and the gold foil (Fig. 2B).

Removal of fixation screws and gold foil continued with resection of the ankylosis mass used a bur, 20 mm-wide gap is created (Fig. 5A). The first attempt to open the mouth reached 15 mm only. A submandibular incision was performed as an access for the hyperplasized coronoid process excision and costochondral graft placement. Following the gap making and coronoidectomy, the patient could open her mouth up to 20 mm.

The costochondral graft from the 6th costa with 5 cm–long graft (4 cm costa and 1 cm chondral) was taken by the orthopedic surgeon. The chondral part of the graft was reshaped to form a convex surface resembling the mandibular condyle. The costochondral graft was then fixed to the ramus (Fig. 5C) using two 6 mm-long screws where the chondral side faced cranially.

The temporalis fascia flap was then made with the size of 2 cm wide and 5 cm long, then the flap was rotated and inserted into the gap around the glenoidal fossa or above the chondral surface of the costochondral graft. The edge of the flap was then sutured to the soft tissue around the fixed costochondral graft (Fig. 5D). A vacuum drain was placed around the costochondral graft.

### 2.3 Postoperative evaluation

**Fig. 6:**
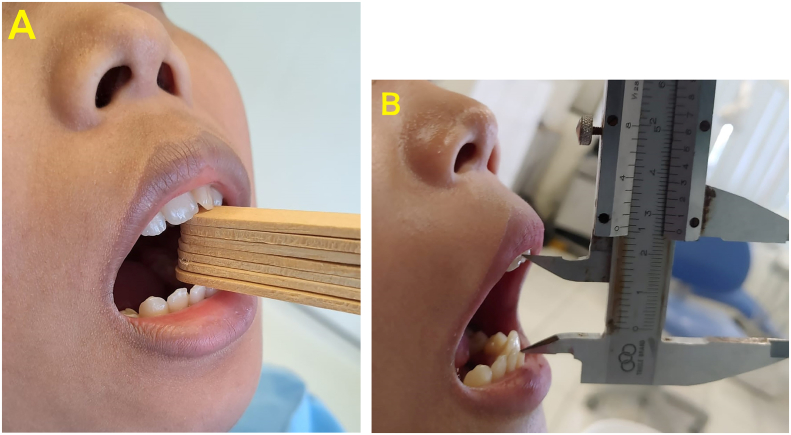
A Postoperative open-mouth practice using ice cream sticks, B Maximum mouth opening reached 26 mm 13 months after surgery.

Seven days postoperatively extraoral edema was still visible, sutures were intact and there was no bleeding nor signs of facial nerve injury. Patient complaint of pain during mouth opening. Individual normal occlusion and no open bite on the contralateral side were found. The patient was instructed to use more ice cream sticks gradually while practicing to maximize mouth opening (Fig. 6A). 3D CT evaluation on the costochondral graft was well fixed to the mandibular ramus.

One month postoperatively showed that extraoral condition found no edema in the surgical area nor complaints of pain, and the surgical wound was healing well. No signs of injury were found in the facial nerve. Individual normal occlusion and no open bite on the contralateral side were found. Mouth opening widen up to 22 mm and no pain. The patient was instructed to keep practicing mouth opening using ice cream sticks.

**Fig. 7:**
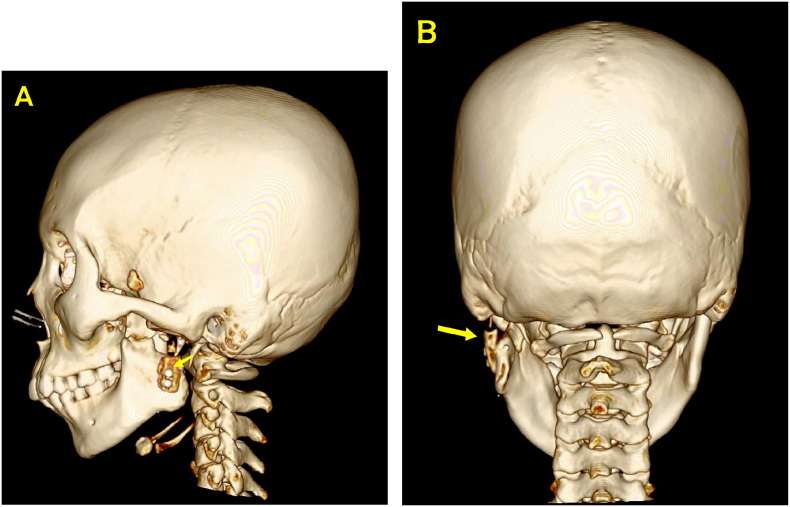
A and B 3D CT image of the head 3 months postoperatively. It showed the costochondral graft was well fixed on the ramus (yellow arrow). (For interpretation of the references to colour in this figure legend, the reader is referred to the web version of this article.)

Three months postoperatively mouth opening widen up to 24 mm and no pain. The patient was instructed to continue practicing mouth opening using ice cream sticks. 3D CT evaluation showed that the costochondral graft was well fixed on the mandibular ramus (Fig. 7).

Eight months postoperatively no increase in mouth opening. Individual normal occlusion and no open bite on the contralateral side were found. The patient was instructed to keep practicing mouth opening using ice cream sticks.

Evaluation on 13 months postoperatively found that extraoral condition was within normal limits and there was scarring in the incision marks. Mouth opening widen up to 26 mm and no pain (Fig. 6B). Individual normal occlusion and no open bite were found on the contralateral side. The work has been reported in line with the SCARE criteria [10].

## 3 Discussion

This case report described a TMJ reankylosis that occurred 5 years after interpositional arthroplasty using gold foil in a 17 years old female, where the first surgery was performed at the age of 12 years. Currently, the use of gold as an interpositional arthroplasty material is very rarely used. Unfortunately, the patient's parents refused the use of a costochondral graft as the interpositional arthroplasty material in the first surgery. The reason for choosing gold foil as an interposition material in first surgery was because it was affordable and easily obtained.

Reankylosis is a complication of TMJ arthroplasty caused by many factors. Particularly in childhood, age is presumably the main factor for reankylosis to occur because bone growth and remodeling are remarkably active, including mandibular bone [5,6]. In this case, the first surgery was performed when the patient was 12 years old. During this age, accelerated growth occurs, and it may result in a narrow and even disappeared gap due to growth in the length of the mandibular ramus. It was proven that in the second surgery showed the gap had disappeared and it was filled by bone and so was part of the gold foil (Fig. 2B). The acceleration of mandibular growth takes place together with the growth of body height, which peaks at the age of 12–14 years in female [15,16]. Besides, children are generally uncooperative or disobey with post-operative instructions. As a result, treatment results are not optimal. This fact was also found by Kaban et al. [6] Furthermore, Hegab [7] stated that the effects of the surgery itself can cause reankylosis, such as inadequate gap width, hematoma and bone dusts when creating the gap which can trigger osteoprogenitor cells to form new bone. The surgical technique used may also become a triggering factor of reankylosis. The author did a literature analysis (Table 1) and concluded that using interpositional material has a lower risk of reankylosis than performing gap arthroplasty only [4,[11], [12], [13], [14]].

**Table 1:** A number of cases of reankylosis with various surgical techniques in 2009–2021.

Author	Year	Number of cases	Age (Year)	Treatment	Eval (Year)	Pre-op MMO (mm)	Post-op MMO (mm)	Reank	% Reank
Zhi et al.	2009	25	22.25	GA	1	7	25.58	3	7.14
		17	22.25	IA + TF, IA + CCG	1	9	29.57	0	0
Elgazzar	2010	11	NO	GA	8	NO	29.1	2	18.2
		14	NO	IA + TF	8	NO	30.7	1	7.1
Babu et al	2013	15	7–29	IA + TF	3	0–2	30–40	0	0
Anchila et al.	2018	76	5 - >20	GA	1	NO	NO	19	25
		115	5 - >20	IA + TF	1	NO	NO	3	2.6
		76	5 - >20	IA + TF+ CCG	1	NO	NO	9	11.8
Gupta et al	2021	16	17.4	IA + TF	1	7.5	35.4	0	0

GA: Gap arthroplasty; IA + TSSF: Interpositional arthroplasty with temporalis flap; IA + CCG: Interpositional arthroplasty with costochondral graft; NO: unknown; Reank: reankylosis; Eval: evaluation.

In this case, the second surgical procedure performed when the patient was 17 years old, through aggressive excision, coronoidectomy, joint lining with temporalis fascia and RCU reconstruction using a costochondral graft accordance with the TMJ ankylosis treatment protocol as proposed by Kaban et al. [6] The use of a costochondral graft is recommended for children because it can grow along with the growth of the child's facial bones and it anatomically resembles the mandibular condyle [6]. In addition, the expensive cost of a TMJ prosthesis, prevention of decrease in mandibular height and openbite occlusion was the reason for using a costochondral graft in this patient's case. The use of costochondral grafts as an interpositional arthroplasty material in adult patients showed good treatment results as reported by El-Sayed and Ahmed et al. [17,18]

The left coronoid process showed hyperplasia (Fig. 3B and 4B) therefore an excision was made on the left coronoid process. Hyperplasia of the coronoid process could result from the effects of TMJ ankylosis itself. During the occurrence of ankyloses, the structures around the TMJ try to maintain normal function through forced mouth opening. As a result, it triggers an inflammatory response and chronic hyperemia in the temporalis muscle tendon, which leads to hyperplasia of the coronoid process [19,20].

The main function of an interpositional material is to prevent contact between two bony surfaces of the joint and avoid recurrence [21]. The temporalis fascia flap has been widely used as interpositional artroplasty material. This material has advantages such as autogenous nature, proximity to the TMJ and hidden scar in the scalp [22]. In addition, according to Khanna and Ramaswami [23], fascia can act as a lubricant that makes movement frictionless.

In this case, the effort to widen the mouth opening after surgery was by using mouth opening exercises assisted by using an ice cream sticks [24]. The maximum mouth opening (MMO) was increased by 6 mm for 1 year with the help of mouth opening exercises using ice cream sticks. There was no reduction in the width of the mouth opening during the 1-year evaluation and radiographic evaluation showed that 3 months after surgery the costochondral graft was well fixed, there were no fractures and resorptions which were complications of the use of the costochondral graft. Evaluation 1 year after surgery there were no complaints of pain and the patient could chew comfortably. The bite or occlusion was within normal limits and no open bite was found on the contralateral side.

## 4 Conclusion

Growing age increases the risk of TMJ reankylosis. Interpositional arthroplasty which used temporalis fascia and costochondral graft followed by unilateral coronoidectomy has resulted in a sufficient mouth opening, improvement of masticatory function. Recurrence was not found in 1 year post-operative evaluation.

## Informed consent

Informed and written consent was obtained for treatment and publishing of photographs.

## Ethical approval

This case report doesn't require ethical approval based on our research ethics committee's institution. Our institution's ethics committee confirmed that this report aligns with routine clinical practice and doesn't involve experimental interventions or additional data collection.

## Funding

This research did not receive any specific grant from funding agencies in the public, commercial, or not-for-profit sectors.

## Author contribution

Conceptualization: P.S.R., P.H.R, M.B.L.

Data Curation: P.S.R., P.H.R, M.B.L.

Formal Analysis: P.S.R., P.H.R, M.B.L.

Funding acquisition: Not applicable.

Investigation: P.S.R., P.H.R, M.B.L.

Project Administration: P.S.R., P.H.R, M.B.L.

Resources: P.S.R., P.H.R, M.B.L.

Software: Not applicable.

Supervision: P.S.R., P.H.R, M.B.L.

Validation: P.S.R., P.H.R, M.B.L.

Visualization: P.S.R., P.H.R, M.B.L.

Writing-original draft preparation: P.S.R., P.H.R, M.B.L.

Writing-review and editing: P.S.R., P.H.R, M.B.L.

All authors have read and agreed to the published version of the manuscript.

## Guarantor

Poerwati Soetji Rahajoe.

## Conflict of interest statement

The authors declare no conflict of interest.

## Data Availability

The data presented in this study are available on request from the corresponding author.
